# Brown adipose tissue uptake of triglyceride-rich lipoprotein-derived fatty acids in diabetic or obese mice under different temperature conditions

**DOI:** 10.1186/s13550-020-00701-6

**Published:** 2020-10-21

**Authors:** Andreas Paulus, Natascha Drude, Wouter van Marken Lichtenbelt, Felix M. Mottaghy, Matthias Bauwens

**Affiliations:** 1 Department of Radiology and Nuclear Medicine, NUTRIM School for Nutrition and Translational Research in Metabolism, Maastricht University Medical Center, Maastricht, The Netherlands; 2 Department of Nuclear Medicine, University Hospital RWTH Aachen, Aachen, Germany; 3 Department of Medical Imaging, Division of Nuclear Medicine, MUMC, Maastricht, The Netherlands; 4 Department of Nanomedicine and Theranostics, Institute for Experimental Molecular Imaging, Uniklinik RWTH Aachen and Helmholtz Institute for Biomedical Engineering, Aachen, Germany; 5 Department of Nutrition and Movement Sciences, NUTRIM School for Nutrition and Translational Research in Metabolism, Maastricht University Medical Center, Maastricht, The Netherlands; 6 Research School NUTRIM, Maastricht University, Universiteitssingel 50, 6229 ER Maastricht, The Netherlands

**Keywords:** Brown adipose tissue, PET, Chylomicron-like particle

## Abstract

**Background:**

In vivo imaging of glucose analogue 2-deoxy-2-[^18^F]fluoro-d-glucose ([^18^F]FDG) via positron emission tomography (PET) is the current gold standard to visualize and assess brown adipose tissue (BAT) activity. However, glucose metabolism is only a part of the metabolic activity of BAT. [^18^F]FDG-PET has been shown in clinical trials to often fail to visualize BAT under insulin-resistant conditions associated with aging and weight gain. We employed a novel developed triglyceride-based tracer to visualize BATs metabolic activity under different temperature conditions as well as under diabetic and obese conditions in preclinical models.

**Results:**

[^18^F]BDP-TG-chylomicron-like particles visualized BAT in control, streptozocin-induced diabetes and obese mice. Increased BAT tracer uptake was found in control mice acutely exposed to cold but not in cold-acclimated animals. Diabetes did not remove BAT tracer uptake, but did limit BAT tracer uptake to levels of control mice housed at 21 °C. In obese animals, BAT tracer uptake was significantly reduced, although the stimulating effect of cold exposure could still be noted.

**Conclusion:**

BAT was visualized in control, diabetic and obese conditions. Streptozocin-induced diabetes, but not obesity, inhibited the stimulatory effect of cold exposure.

## Background

Brown adipose tissue (BAT) research has evolved rapidly within the last 2 decades to an important field in endocrine research. BAT has the ability to uncouple its ATP production and to produce heat instead of ATP. During this process, protons enter the mitochondrial matrix by uncoupling protein 1 (UCP1) [1], a BAT and beige fat specific protein, and release their energy as heat [2, 3]. This process is also known as nonshivering thermogenesis [2, 4].

During cold exposure, thermoreceptors in the skin are stimulated and activate neurons in the hypothalamus, resulting in a release of norepinephrine [2, 5]. Norepinephrine binding to β3-adrenoreceptors on brown adipocytes activates a signaling cascade, which will lead to lipolysis of intracellular triglycerides (TGs) and at the end result in UCP1 activation [6, 7]. Therefore, BAT might be an interesting target in the fight against obesity as it “burns” lipids instead of storing them.

To visualize BAT and its metabolism, noninvasive imaging is a central technique. BAT activity is triggered by cold stimulation and this increases the chance to visualize BAT in animals and lean humans [8–10]. Numerous visualization and quantification techniques for BAT exist [11, 12] but most BAT imaging studies have been performed with 2-deoxy-2-[^18^F]fluoro-d-glucose ([^18^F]FDG) positron emission tomography (PET). Before the introduction of hybrid PET/CT systems, symmetrical accumulations in the supraclavicular area in [^18^F]FDG scans (e.g., during PET scans acquired in patients with cancer) were attributed to cervical muscle until later scans using PET/computed tomography (CT) could show that Hounsfield units corresponded them of adipose tissue [13–15]. Therefore, a high number of retrospective BAT studies have been published and until now [^18^F]FDG is used to visualize BAT. The prevalence to find active BAT depots during [^18^F]FDG/PET scans is strongly dependent on different variables such as age, BMI or outdoor temperature [16, 17].

Even though BAT [^18^F]FDG/PET scans provide valuable information, they suffer from two shortcomings: (1) Fatty acids (FAs) have been identified as the main metabolized substance class [18, 19] and therefore [^18^F]FDG/PET might underestimate BATs metabolic activity. (2) In clinical studies, [^18^F]FDG uptake in BAT was impaired in diabetic patients but uptake of [^18^F]FTHA (a radiolabeled FA) was not altered when compared to non-diabetic controls [20]. As a high number of obese patients suffer from diabetes type II [21], their insulin resistance will additionally decrease [^18^F]FDG uptake in BAT and underestimate BATs total metabolic activity [22, 23].

Due to these facts, BAT visualization with lipid tracers could provide additional information on the lipid-metabolism of BAT, next to the glucose-metabolism as provided by [^18^F]FDG. FA-based tracers, such as [^18^F]FTHA [24] or [^125^I]BMIPP [25], have been developed and can be found in clinical applications. But even FA tracer might not be the optimal choice.

During BAT activation, internal lipid droplets are replenished by nutrient uptake from plasma in three different ways: uptake of FAs from TG-rich lipoproteins (TRL) after external lipolysis, glucose uptake followed by de novo lipogenesis and uptake of circulating albumin-bound FAs [2, 26–28]. It was found that TRL-derived FAs are the main supply of TGs in BAT [28, 29]. Due to this fact, free radiolabeled FAs applied in vivo rely on many different factors, like uncontrolled uptake and incorporation processes before they are taken up and eventually metabolized by BAT, which we wanted to avoid. Therefore, we have developed a radiolabeled TG [30] which we were able to incorporate into a pre-synthesized chylomicron-like particle. Additionally, the tracer was tested in vivo and was able to visualize BAT at room temperature (21 °C) conditions and its uptake was increased by acute cold exposure [31].

In this manuscript, we investigate the effect of induced diabetes and obesity as well as acute cold and cold acclimation in a mouse model on BAT metabolism visualized by our recently developed lipid-based tracer [^18^F]BODIPY ([^18^F]BDP)-TG-chylomicron-like particle ([18F]BDP-TG). Our hypothesis is that [18F]BDP-TG has discriminatory power in these models and allows to assess BAT metabolic activity—not as a replacement for the widely used [^18^F]FDG but as a tool providing additional information.

## Methods

Commercially available compounds were used without further purification unless otherwise stated. BDP-FA was purchased from Thermo Fischer Scientific (99%) (Netherlands). 1,2-diolein was purchased from Cayman Chemicals (USA) (≥ 95%). All HPLC purifications (1.0 mL/min, solvent A; 0.1% TFA in H_2_O, solvent B; CH_3_CN, 50 °C) were performed on a Shimadzu UFLC HPLC system equipped with a DGU-20A_5_ degasser, a SPD-M20A UV detector, a LC-20AT pump system, a CBM-20A communication BUS module, a CTO-20AC column oven and a Scan-RAM radio-TLC/HPLC-detector from LabLogic using an Aeris™ Widepore column (C4, 3.6 μm, 4.6 mm × 250 mm) for the BODIPY-triglyceride (BDP-TG). ESI–MS was performed on an Applied Biosystems SCIEX API 150 EX electrospray ionization quadrupole (ESI-Q) mass spectrometer with the method of McAnoy et al. [32]. Briefly, 0.1 M aqueous ammonium acetate solution was added to the sample to observe the ammonium salt of the synthesized TG in the MS.

^1^H-NMR spectra were carried out on a Bruker Ultrashield*TH 400 plus* at 400 MHz. Tol-d_8_ was used as solvent with TMS as internal standard. Chemical shifts are reported in parts per million (ppm) relative to the internal standard.

### Synthesis of chylomicron-like particles

Synthesis of chylomicron-like particles was performed as reported before [33, 34]. Briefly, emulsion particles were prepared from triolein (70 mg), egg yolk phosphatidylcholine (Lipoid) (22.7 mg), lysophosphatidylcholine (2.3 mg), cholesteryl oleate (3.0 mg) and cholesterol (2.0 mg). Sonification was performed using a Soniprep 150 (MSE Scientific Instruments, UK) that was equipped with a water bath for temperature (54 °C) maintenance, at 10 μm output. The emulsion was fractionated by density gradient ultracentrifugation steps in a Beckman SW 40 Ti rotor. After centrifugation for 30 min at 17,850 rpm at 20 °C, an emulsion fraction containing chylomicron-like particles was removed from the top of the tube by aspiration. Characterization of chylomicron-like particles was done by DLS and transmission electron microscopy [31]. Chylomicron-like particles were stored at 4 °C and were used within 5 days following preparation.

### Synthesis of BDP-TG

Synthesis was performed as reported before [30]. Briefly, BDP-FL-C_16_ (300 μg, 0.6 μmol) in acetonitrile was evaporated to complete dryness before the reactant was reconstituted in toluene (100 μL). To the resulting solution SOCl_2_ in toluene (100 μL, 4 vol%) was added, incubated for 5 min at 70 °C in a closed vial and evaporated. The product was reconstituted in toluene (50 μL) containing 1,2-diolein (2 μL, 2.8 μmol) and heated to 100 °C for 30 min. After the reaction time, purification by HPLC (1 mL/min, 30–15% A in 5 min, 15% to 0% A from 5 to 6 min, 0% A to 20 min) yielded **2** (225 μg, 75%) as a red solid; retention time (t_R_) = 12.3 min. ESI–MS (+) m/z (%) = 1058 (100) [M—F^−^]^+^, 1095 (82) [M + NH_4_]^+^. ^1^H NMR (400 MHz, Tol-d_8_); δ (ppm) = 5.46 (m, 4H), 4.26 (m, 2H), 4.06 (m, 2H), 3.13 (m, 1H), 1.75 (s, 3H) [30].

### Radiolabeling of BDP-TG

Radiolabeling was performed as reported before [30]. Briefly, aqueous fluoride-18 solution was loaded on a QMA-cartridge which was preconditioned with 15 mL K_2_CO_3_ in H_2_O and 20 mL H_2_O. Fluoride-18 (42 MBq) was eluted with a mixture of 600 μL acetonitrile, 400 μL H_2_O and 6 mg Sodium *p*-toluenesulfonate (Sigma-Aldrich). Fluoride-18 solution was transferred into a drying vessel containing tetra-*n*-butylammonium bromide (80 μL) as a phase transfer agent. Acetonitrile (3 × 1.0 mL) was added and the solution of fluoride-18 was dried by heating to 100 °C with a continuous flow of argon. After reconstitution of Fluoride-18 in anhydrous acetonitrile (100 μL), a solution of BDP-TG in toluene (107 μg, 0.1 μmol in 50 μL) and SnCl_4_ (0.2 M in acetonitrile, 100 μL) was added to the solution with the activity and the reaction solution was stirred at room temperature (r.t.) for 30 min.[^18^F]BDP-TG was obtained (decay corrected radiochemical yield (RCY): 60%, 25 MBq) with a decay corrected specific activity of 250 MBq/μmol and a radiochemical purity of 45% determined by a radio-TLC with toluene, CHCl_3_ and MeOH (80.9%, 14.3%, 4.8%) of the reaction solution.

### Ex vivo incorporation of [^18^F]BDP-TG into chylomicron-like particles

Incorporation of radiolabeled [^18^F]BDP-TG was performed as reported before [31]. Briefly, the [^18^F]BDP-TG solution (233 MBq) was quenched with 500 μL H_2_O and centrifuged for 5 min. The organic phase was washed 3 times with 500 µL H_2_O before [^18^F]BDP-TG was reconditioned in 20 μL EtOH.[^18^F]BDP-TG could be obtained with a radiochemical purity of > 96% and an overall decay corrected radiochemical yield of 21%. 400 μL chylomicron-like particles in HEPES were added (1.5 mg TG content) and incubated for 1 h at r.t..[^18^F]BDP-TG-chylomicron-like particles were obtained (overall decay corrected RCY: 18%, 19 MBq) with a radiochemical purity of > 99% analyzed by gel electrophoresis and radio-TLC.

### Animal experiments

Experimental protocols were approved by the “Centrale Commissie Dierproeven” (local animal welfare ethics commission) and all animal experiments and procedures were performed in accordance with the guidelines set of by this institution. Forty-eight female C57Bl/6 mice were divided into three groups. Group 1 served as control group and only received a standard diet (D12450B, 10% kcal fat, Research Diets Inc.). Group 2 was injected with streptozocin 10 days before the experiment to destroy the β-cells in the pancreas [35] and from there on received a high-fat diet (D12451, 45% kcal fat, Research Diets Inc.). An animal was considered as diabetic and included in the study only when the glucose level at the day of the experiment was > 10 mM. Group 3 received the high-fat diet (45% kcal fat, Research Diets Inc.) over a period of 16 weeks.

**Fig. 1:**
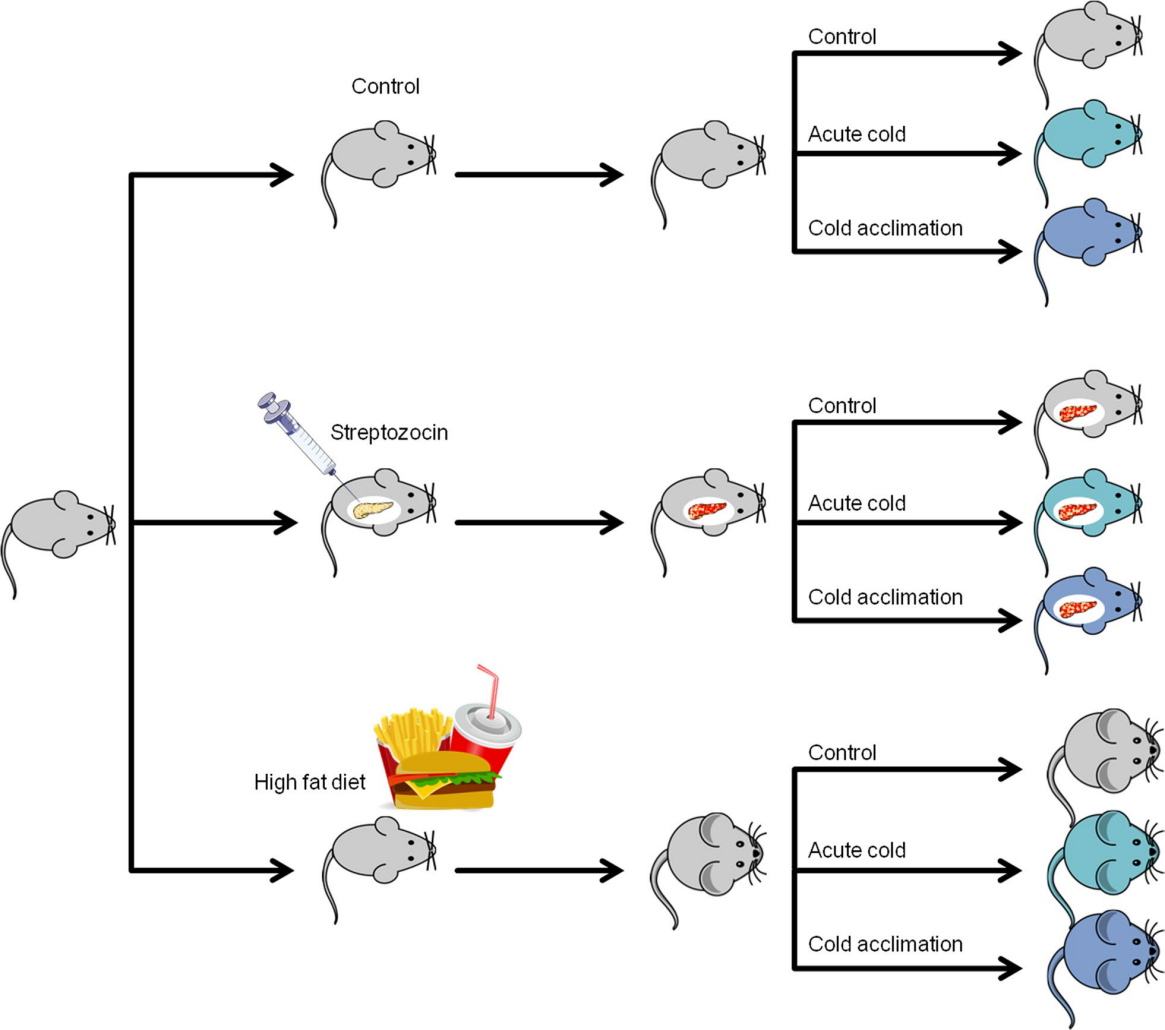
Mice were divided into 3 groups. Group 1 was used as a control group. Group 2 was injected with streptozocin 10 days before the imaging experiment and afterward fed with a high-fat diet to induce diabetes. Group 3 received a high-fat diet over 16 weeks. All groups were divided into 3 subgroups where subgroup 1 was housed at 21 °C before the experiment. Subgroup 2 was housed at 21 °C but was exposed to 6 °C for 4 h prior to tracer injection. Subgroup 3 was exposed to cold for 6 h per day for 28 days before the imaging experiment

All groups were divided into 3 subgroups: subgroup 1 was fasted for 4 h at 21 °C prior the imaging experiment; subgroup 2 was housed at 21 °C but was fasted for 4 h during exposure to 6 °C prior to tracer injection; subgroup 3 was exposed to cold for 6 h per day for 28 days and was fasted for 4 h before tracer injection during the afternoon (Fig. 1).

Mice were anesthetized (pentobarbital, 60 mg/kg i.p.), and injected with [^18^F]BDP-TG-chylomicron-like particles (1–10 MBq) in HEPES (100 μL) via the tail vein. Mice were scanned dynamically for 32.5 min on a microPET (Focus 120, Siemens). Images were analyzed using Pmod V3.707. After the scanning time animals were killed and organs harvested, weighed wet and counted using a WIZARD^2^ automatic γ-counter from PerkinElmer.

### Statistical analyses

Data are presented as mean ± standard deviation (SD), unless indicated otherwise. Two-way ANOVA was used to test for differences between all groups, while a two-tailed Mann–Whitney U test was used for comparing two specific groups. Differences at a probability level (*p*) of 0.05 were considered statistically significant. GraphPad Prism 5.01 (La Jolla, CA, USA) for Windows was used for statistical analyses.

## Results

### Synthesis of BDP-TG, radiolabeling and incorporation into chylomicron-like particles

Esterification of BDP-C_16_ yielded BDP-TG (45 ± 8%) after HPLC purification (t_r_ 12.3 min) and its identity was confirmed by NMR and ESI–MS like reported before [30]. Radiolabeling of BDP-TG was conducted as described before (44% decay corrected rcy., 250 MBq/μmol corrected specific activity). After washing steps, [^18^F]BDP-TG could be obtained with an overall RCY of 21% (radiochemical purity > 96%). Due to its lipophilicity and the numerous necessary washing steps, the overall rcy. decreased by half during this procedure.

Chylomicron-like particles were synthesized with a mean diameter of 164 ± 20 nm and a polydispersity index of 0.181 (n = 4). Electron microscopy confirmed the identity of the particles [31].

[^18^F]BDP-TG was incorporated into chylomicron-like particles like reported before [31]. After 60 min at room temperature, more than 99% of the [^18^F]BDP-TG was incorporated in the particle and no free fluoride-18 was found in solution.

### Animal experiments

[^18^F]BDP-TG-chylomicron-like particles (1–10 MBq) were injected *i.v.* into female C57Bl/6 mice, which were fasted for 4 h either at 21 °C or at 4 °C. After scanning for 32.5 min, the animals were euthanized and the organs were harvested. Subgroups of animals consisted of at least 3 animals per group (Table 1).

**Table 1:** Number of animals and weight in groups and subgroups±

Number of animals			
Start of experiment	Control	Diabetic	Obese
Room temperature	4	6	6
Acute cold	4	6	6
Cold acclimated	4	6	6

Included in study	Control	Diabetic (*)	Obese (+)
Room temperature	4	4	5
Acute cold	4	4	5
Cold acclimated	4	3	6

Weight (start experiment)	Control	Diabetic	Obese
Room temperature	19.3 ± 0.7	19.3 ± 0.5	19.0 ± 0.9
Acute cold	19.3 ± 0.7	19.2 ± 0.7	19.7 ± 0.5
Cold acclimated	19.8 ± 0.7	19.5 ± 0.5	20.2 ± 0.4

Weight (end of experiment)	Control	Diabetic	Obese
Room temperature	20.5 ± 1.0	17.7 ± 1.6	24.6 ± 1.4
Acute cold	20.8 ± 0.2	18.7 ± 0.7	22.4 ± 1.8
Cold acclimated	20.3 ± 1.2	18.6 ± 0.6	23.6 ± 0.5

(*) Drop-out caused by insufficiently developed hyperglycemia (n = 5) or reaching of humane endpoint due to combination of cold exposure and hypoglycemia shortly after streptozocin administration (n = 2)

( +) Drop-out caused by equipment failure

**Fig. 2:**
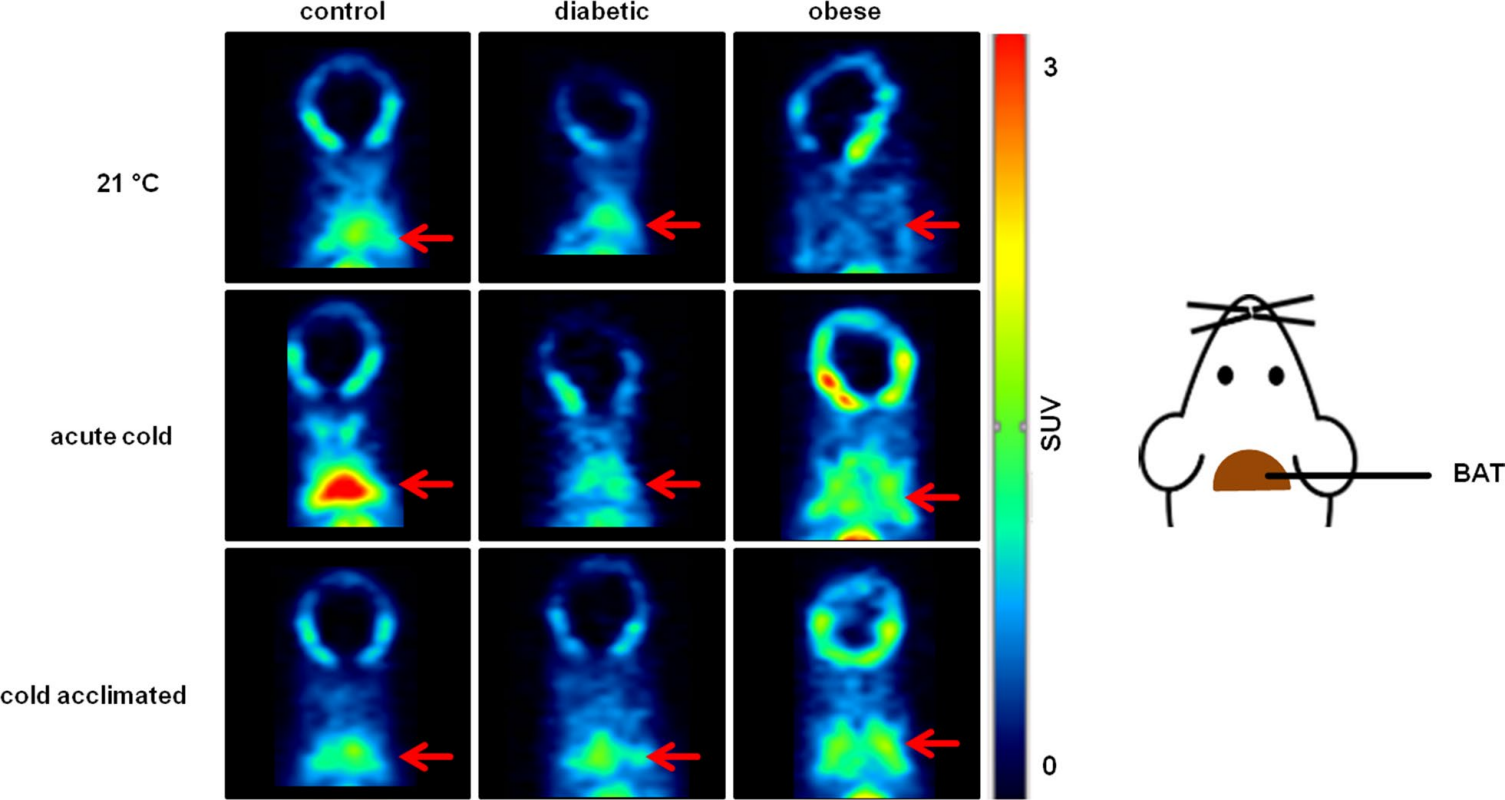
PET images (22–32.5 min) of [^18^F]BDP-TG- chylomicron-like particles in control, diabetic and obese mice under different temperature conditions

Analysis of the PET images showed highest uptake in liver, and heart of mice exposed at 21 °C, acute cold and cold acclimation in control, diabetic and obese animals (Fig. 2 and Additional file 1: Fig. S1a). A rapid increase with a slow washout in both organs could be visualized (Additional file 1: Fig. S1a). In bone, a constant increase in signal was observed (Additional file 1: Fig. S1b), which probably indicates a defluorination process of the tracer in vivo, as reported in the literature [36]. Lung showed a fast increase with a fast washout and stayed constant at later time points under all temperature conditions. Brain as a negative control showed negligible uptake (Additional file 1: Fig. S1b).

BAT tracer uptake was significantly increased under control conditions by acute cold versus 21 °C housed animals (*p* < 0.01) as well as by acute cold versus cold acclimation (*p* < 0.01) (Table 2). Diabetic animals did not show any significant response to acute cold exposure or cold acclimation. In obese animals, acute cold increased BAT tracer uptake significantly versus 21 °C housed animals (*p* < 0.001). Also increased uptake values were found in cold-acclimated animals versus animals housed at 21 °C (*p* < 0.001) and in acute cold animals versus cold-acclimated animals (*p* < 0.001).

**Table 2:** SUV_mean_ values of BAT at 30 min p.i. (expressed as mean ± SD)

	Control			Diabetic			Obese		
	21 °C	Acute cold	Cold accl	21 °C	Acute cold	Cold accl	21 °C	Acute cold	Cold accl
SUV-mean of BAT	1.06 ± 0.13	1.81 ± 0.68	1.02 ± 0.09	0.99 ± 0.13	1.08 ± 0.06	1.01 ± 0.09	0.71 ± 0.13	1.36 ± 0.23	1.00 ± 0.07

In animals housed at 21 °C, BAT tracer uptake was significantly higher in control animals versus obese animals (*p* < 0.001). Under acute cold exposure, the control group still showed significantly increased BAT tracer uptake versus the obese group (*p* < 0.05). BAT tracer uptake was significantly increased in the control group exposed to acute cold versus the diabetic group (*p* < 0.01). In animals housed at 21 °C, BAT tracer uptake was significantly higher in diabetic animals versus obese animals (*p* < 0.001) but when these groups were exposed to acute cold obese animals showed a significantly higher BAT tracer uptake versus diabetic animals (*p* < 0.01).

**Fig. 3:**
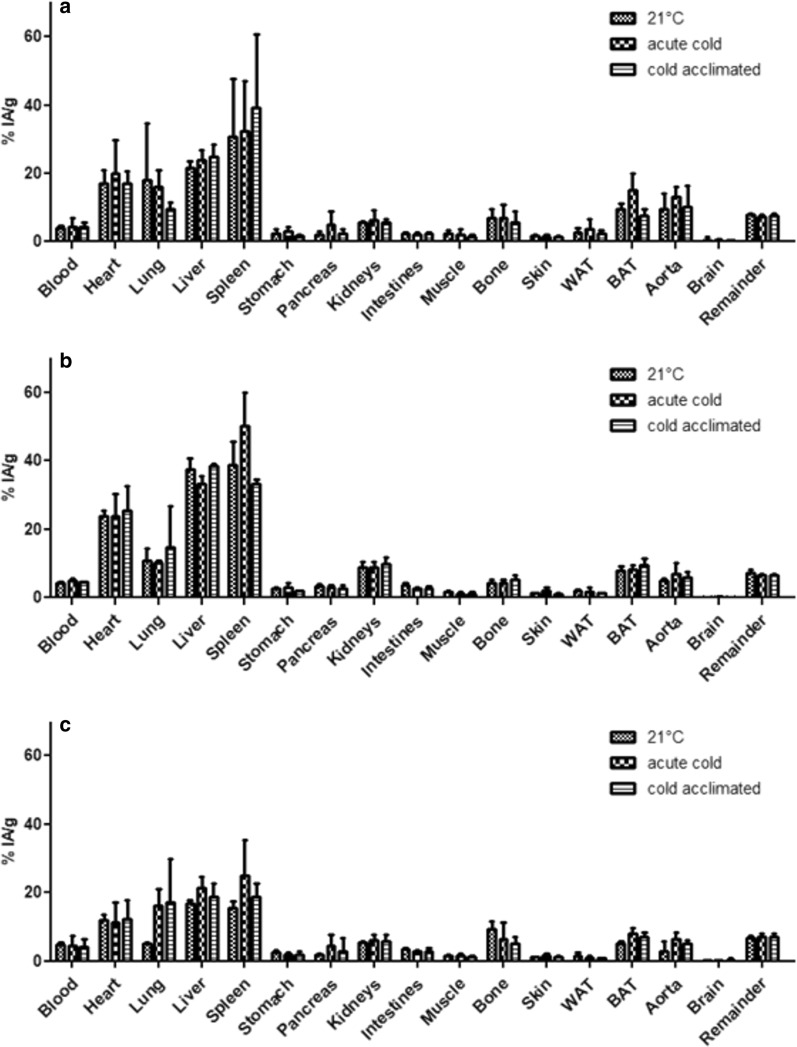
Biodistribution data of [^18^F]BDP-TG-chylomicron-like particles in control (**a**) diabetic (**b**) and obese (**c**) mice at room temperature, under acute cold and cold acclimation 30 min after injection of [^18^F]BDP-TG-chylomicron-like particles. For indication of significance, see Table 3

PET images are supported by the results of the biodistribution. Highest uptake values in all groups and subgroups were found in spleen, liver, lung and heart (Fig. 3 and Table 3). After 30 min, only minor tracer amounts were found in the blood, indicating a fast blood clearance during the scanning time. In all conditions, uptake by BAT was significantly higher compared to WAT (Fig. 3 and Table 3).

**Table 3:** Biodistribution data of control, diabetic and obese mice at room temperature, under acute cold and cold acclimation 30 min after injection of [18F]BDP-TG-chylomicron-like particles, expressed as %IA/g

	Control			Diabetic			Obese		
	21 °C (%)	Acute cold (%)	Cold accl (%)	21 °C (%)	Acute cold (%)	Cold accl (%)	21 °C (%)	Acute cold (%)	Cold accl (%)
Blood	3.9 ± 0.7	4.5 ± 2.6	4.3 ± 1.4	4.4 ± 0.3	4.9 ± 0.6	4.5 ± 0.3	4.9 ± 0.7	4.6 ± 2.9	4.1 ± 2.3
Heart	16.9 ± 4.1	19.9 ± 9.8	17.2 ± 3.3	23.7 ± 1.9	23.8 ± 6.6	25.5 ± 7.2	12.1 ± 1.7	11.4 ± 5.8	12.1 ± 5.7
Lung	18.0 ± 16.8	16.0 ± 5.0	9.7 ± 1.7	10.7 ± 3.9	10.2 ± 0.7	14.7 ± 12.1	5.2 ± 0.3	16.0 ± 4.9	17.0 ± 13.0
Liver	21.5 ± 2.0	23.7 ± 3.0	24.9 ± 3.6	37.4 ± 3.3	33.4 ± 2.1	38.4 ± 0.5	16.7 ± 1.2	21.5 ± 3.3	18.9 ± 3.9
Spleen	30.8 ± 16.9	32.3 ± 14.8	39.3 ± 21.3	38.7 ± 6.9	50.3 ± 9.7	33.4 ± 1.2	15.5 ± 1.9	24.8 ± 10.5	18.9 ± 3.7
Stomach	2.3 ± 1.3	3.2 ± 1.1	1.6 ± 0.3	2.7 ± 0.2	2.9 ± 1.5	1.9 ± 0.1	2.4 ± 0.7	2.0 ± 0.5	1.7 ± 1.1
Pancreas	2.2 ± 1.0	5.1 ± 3.9	2.3 ± 1.4	3.2 ± 0.8	2.9 ± 0.5	2.8 ± 0.7	1.9 ± 0.3	4.3 ± 3.3	3.0 ± 3.8
Kidneys	5.6 ± 0.5	6.3 ± 3.1	5.7 ± 1.0	9.0 ± 1.5	9.0 ± 1.5	9.9 ± 2.1	5.3 ± 0.5	6.2 ± 1.6	5.6 ± 2.0
Intestines	2.3 ± 0.4	2.2 ± 0.4	2.3 ± 0.5	3.8 ± 0.6	2.8 ± 0.3	2.7 ± 0.7	3.3 ± 0.6	2.7 ± 0.5	2.8 ± 1.0
Muscle	2.4 ± 1.1	2.0 ± 1.6	1.4 ± 0.6	1.6 ± 0.3	1.1 ± 0.6	1.2 ± 0.6	1.5 ± 0.2	1.6 ± 0.6	1.3 ± 0.3
Bone	7.1 ± 2.6	7.1 ± 3.6	5.8 ± 3.2	4.3 ± 0.9	4.4 ± 0.8	5.3 ± 1.3	9.2 ± 2.5	6.5 ± 4.8	5.2 ± 1.9
WAT	2.7 ± 1.4	3.7 ± 2.9	2.4 ± 0.9	1.9 ± 0.4	1.8 ± 1.2	1.4 ± 0.1	1.5 ± 0.9	0.8 ± 0.6	0.7 ± 0.2
Skin	1.6 ± 0.6	1.5 ± 0.7	1.5 ± 0.4	1.3 ± 0.2	1.9 ± 0.9	1.2 ± 0.2	1.1 ± 0.1	1.5 ± 0.5	1.1 ± 0.2
BAT	9.6 ± 1.7	15.1 ± 4.7	7.6 ± 1.9	8.0 ± 1.3	8.2 ± 1.4	9.2 ± 1.8	5.1 ± 0.6	8.0 ± 1.8	6.9 ± 1.4 ‬
Aorta	9.7 ± 4.5	13.1 ± 3.1	10.1 ± 6.4	4.9 ± 0.9	6.8 ± 3.3	6.0 ± 1.5	3.0 ± 2.7	6.5 ± 2.0	5.1 ± 1.0
Brain	0.6 ± 0.7	0.4 ± 0.3	0.3 ± 0.1	0.2 ± 0.0	0.2 ± 0.0	0.2 ± 0.0	0.2 ± 0.0	0.2 ± 0.1	0.4 ± 0.4
Remainder	8.0 ± 0.4	7.3 ± 0.5	7.6 ± 0.8	7.2 ± 1.0	6.6 ± 0.2	6.5 ± 0.3	6.8 ± 0.5	7.0 ± 0.9	6.9 ± 1.0

BAT was the only tissue to be statistically significantly affected by the effect of temperature throughout the study (Tables 3, 4). BAT tracer uptake in control animals exposed to acute cold showed a clear trend toward increased uptake in BAT compared to animals housed at 21 °C but could not reach significance (*p* = 0.07). Acute cold versus cold acclimation animals showed a significant increase in uptake in animals exposed to acute cold (*p* < 0.05) where housing at 21 °C versus cold acclimation did not show significant differences. Other organs did not show any response to acute cold or cold acclimation (two-way ANOVA) (Fig. 3 and Table 3 as well as Table 4). There were multiple organs and tissues where tracer uptake was affected by the metabolic model: liver, kidneys, heart, aorta, spleen, white adipose tissue, brown adipose tissue and the intestines (Table 4).

**Table 4:** Statistical significance of impact of temperature and metabolic model on the biodistribution data of [18F]BDP-TG-chylomicron-like particles

	Significant impact of temperature?	% of variation explained by temperature	*p* value	Significant impact of metabolic model?	% of variation explained by metabolic model	*p* value
Blood	No	1.08	0.86	No	1.14	0.85
Heart	No	0.25	0.93	*Yes*	*50.40*	*< 0.0001*
Lung	No	1.97	0.71	No	1.61	0.76
Liver	No	1.25	0.2063	*Yes*	*84.46*	*< 0.0001*
Spleen	No	4.74	0.29	*Yes*	*37.31*	*0.0005*
Stomach	No	17.69	0.051	No	4.02	0.47
Pancreas	No	10.02	0.21	No	0.17	0.97
Kidneys	No	1.03	0.73	*Yes*	*52.76*	*< 0.0001*
Intestines	No	12.45	0.068	*Yes*	*23.94*	*0.0085*
Muscle	No	7.87	0.26	No	11.92	0.14
Bone	No	4.72	0.42	No	14.16	0.085
WAT	No	3.60	0.45	*Yes*	*31.92*	*0.0032*
Skin	No	10.38	0.17	No	6.75	0.31
*BAT*	*Yes*	*16.05*	*0.0054*	*Yes*	*27.58*	*0.0003*
Aorta	No	8.51	0.11	*Yes*	*41.72*	*0.0002*
Brain	No	0.80	0.88	No	10.42	0.19
Remainder	No	4.53	0.41	*Yes*	*24.01*	*0.015*

BAT tracer uptake in obese animals exposed to acute cold showed significantly increased uptake in BAT compared to animals housed at 21 °C (*p* < 0.05). Additionally, uptake in cold-acclimated animals versus animals housed at 21 °C was significantly increased (*p* < 0.05). No difference could be found in acute cold versus cold-acclimated animals (Fig. 3 and Table 4).

BAT tracer uptake in control, diabetic and obese animals was compared at different temperatures and significantly increased uptake values were found in animals housed at 21 °C at the following conditions: control versus obese (*p* < 0.001) and diabetic versus obese (*p* < 0.01). Acute cold exposed animals showed increased uptake when following conditions where compared: control versus obese (*p* < 0.05) and control versus diabetic (*p* < 0.05). In animals, acclimated to cold only diabetic versus obese animals could show significantly increased uptake values (*p* < 0.05) (Fig. 3 and Tables 3, 4).

**Fig. 4:**
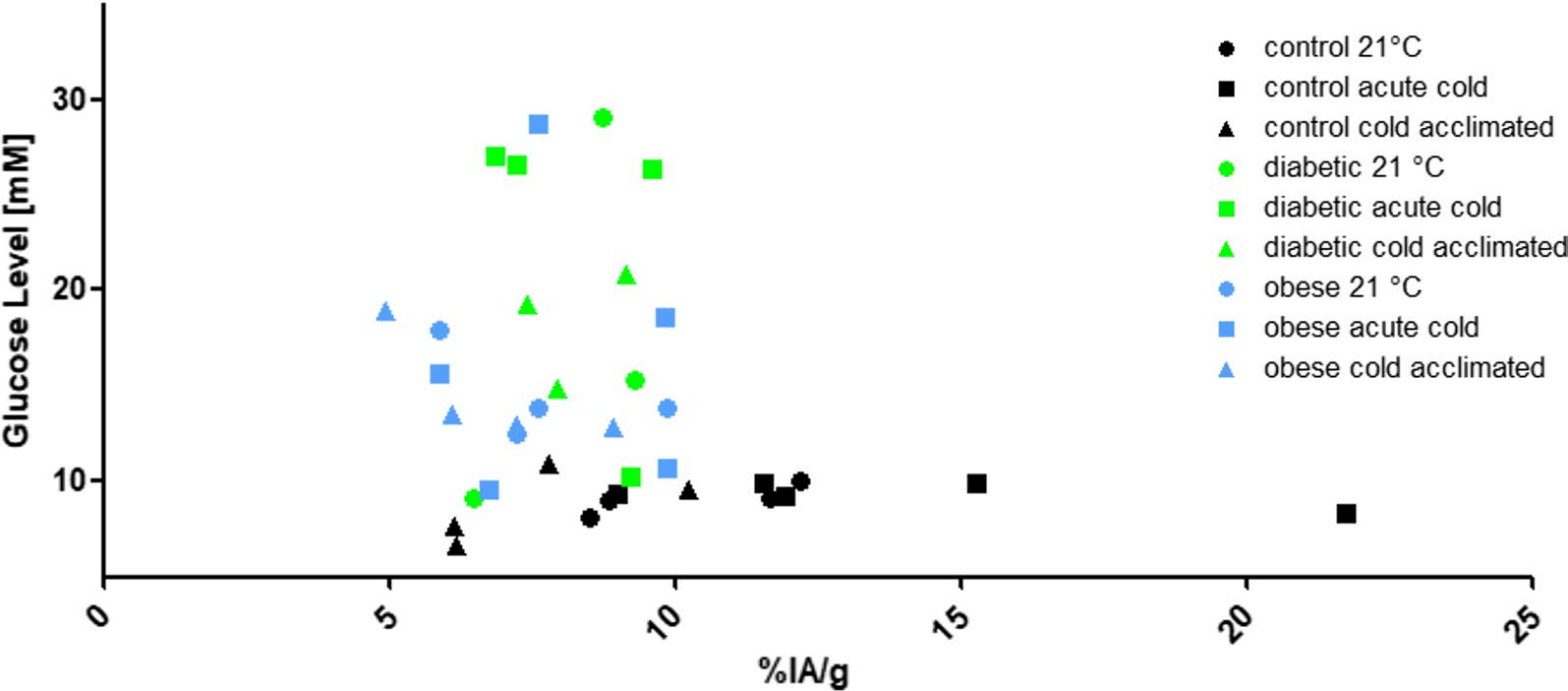
Glucose level [mM] and %IA/g of [^18^F]BDP-TG-chylomicron-like particles uptake in BAT in control and diabetes-induced mice

BAT tracer uptake of [^18^F]BDP-TG was plotted against the glucose level and diabetic and obese animals differed significantly by their glucose levels compared to control animals (Mann–Whitney U, n = 37, two tailed, *p* < 0.05). No difference in BAT tracer uptake values could be found (Fig. 4).

## Discussion

The presented study investigated the impact of different cold exposure protocols on BATs lipid uptake in diabetic and obese animal models. Although the uptake of our recently developed radiotracer was primarily in the liver and to a lesser degree in BAT, we were able to visualize and quantify BATs uptake characteristics under different conditions. An increase in tracer uptake under control conditions due to acute cold exposure was observed but could not reach significance (Fig. 3a). This effect was also observed in human studies where long-term cold acclimation could not further increase [^18^F]FDG uptake in BAT [37]. Nevertheless, in other studies with different cooling protocols, cold acclimation could demonstrate a significant increase in BAT [^18^F]FDG-uptake [17, 38]. Therefore, a comparison of different studies or a translation of the here presented results might still be difficult as long as there is no standardized cooling protocol for BAT imaging in small animals as well as humans.

In diabetic mice, BAT could be visualized by our tracer under conditions where [^18^F]FDG would have failed [22, 23]. In general, BAT tracer uptake was comparable to uptake in control mice housed at 21 °C which indicates BATs metabolic activity under diabetic conditions. However, no difference in BAT tracer uptake between housing at 21 °C, acute cold exposure or cold acclimation could be found. Even the pronounced uptake after acute cold exposure versus 21 °C housing in control animals could not be induced (Fig. 3b). In a recent publication, Heine et al. described BATs dependence on insulin to maintain its lipolytic capacity to process TRLs [39]. The here presented results are in accordance to this work.

These results differ from results reported in the human situation where BATs [^18^F]FDG uptake was increased in diabetes type II patients after cold acclimation [40]. Our diabetic animal model reflects more the situation in diabetes type I which may explain the differences between those studies. Also the fact that mice housed at 21 °C are already under constant thermal stress, as their thermoneutral zone is at ~ 29 °C [41], might influence the results and complicates the comparison to the human situation.

As a high number of patients who suffer from obesity also suffer from type II diabetes, additional experiments with obese but not diabetic animals were performed. A significant reduction of BAT tracer uptake, and therefore a reduction in BATs activity, under control and acute cold conditions was observed in comparison with animals housed at 21 °C. Only in cold-acclimated animals BAT tracer uptake values were comparable to the control group. We hypothesized that through long-term cold exposure, similar to human studies [17, 38], BAT is activated and again takes part in whole-body metabolism. Therefore, these results might reflect the human situation to some parts and might be easier to translate as the results obtained from the (type I) diabetic mice group.

This study is not without its limitations. As a high-fat diet was needed to obtain obese mice, the tracer uptake in these mice may be confounded by the diet-induced increase in plasma lipid concentrations and the increased body weight. The changes due to a high-fat diet have been described in detail by other researchers [42, 43]. Diabetic mice were kept at a high-fat diet as well for the last 10 days prior to imaging in order to allow a correct comparison with obese mice, which makes a direct comparison to control animals however more difficult.

We investigated our recently developed radiotracer in in vivo models [30, 31] using a different sedation protocol of mice (pentobarbital instead of isoflurane). This was necessary since volatile anesthetics, such as isoflurane, were found to have an inhibitory effect on norepinephrine-related thermogenesis. The nonvolatile anesthetics, such as pentobarbital, do not show this antithermogenic effect and seem to be superior in this setting [44]. Therefore, BAT tracer uptake was found to be significantly higher as in our previous study [31].

Uptake in BAT was lower and uptake by liver, spleen and heart were higher in our study when compared to others using [^3^H]-labeled oleate [45]. We believe that the [^18^F]BDP-TG-chylomicron-like particle is not lipolyzed as efficiently as particles labeled with tri[^3^H]oleate, but further research is required to fully understand the uptake mechanism of the tracer. In order to fully understand BAT activity, a head-to-head comparison of [^18^F]BDP-TG-chylomicron-like particle with [^18^F]-FDG would also be beneficial—however, this was outside the scope of the current study.

## Conclusion

We were able to visualize BAT uptake of TRL-derived FAs under different metabolic and temperature conditions. BAT activation by cold exposure under diabetic conditions could not be detected indicating an insulin-dependent uptake mechanism. Obesity in general reduced triglyceride uptake but by cold exposure (acute, acclimated) levels of control animals housed at 21 °C could be retrieved. Together, this indicates an insulin-dependent as well as an insulin-independent uptake mechanism for TRL-derived FAs in BAT in mice—more research is however needed to elucidate these mechanisms.

## Supplementary information

**Additional file 1: Fig. S1**. Time activity curves in control mice housed 21 °C in **a** liver and heart **b** BAT, brain, bone, lung, exemplary for all other groups and sub-groups.

## Data Availability

All data generated or analyzed during this study are included in this published article.

## Abbreviations

[^18^F]FDG: 2-Deoxy-2-[^18^F]fluoro-d-glucose
PET: Positron emission tomography
BAT: Brown adipose tissue
UCP1: Uncoupling protein 1
TGs: Triglycerides
FAs: Fatty acids
TRL: TG-rich lipoproteins
[^18^F]BDP: [^18^F]BODIPY
