# Supplementary material for: Brown adipose tissue uptake of triglyceride-rich lipoprotein-derived fatty acids in diabetic or obese mice under different temperature conditions
Source: EJNMMI Res. 2020 Oct 21;10:127. doi: 10.1186/s13550-020-00701-6 (PMC7578207; doi:10.1186/s13550-020-00701-6)
Supplement: Supplementary file 1 — Additional file 1: Fig. S1. Time activity curves in control mice housed 21 °C in a liver and heart b BAT, brain, bone, lung, exemplary for all other groups and sub-groups. [file 13550_2020_701_MOESM1_ESM.docx]

**Brown adipose tissue uptake of triglyceride-rich lipoprotein derived fatty acids in diabetic or obese mice under different temperature conditions**

Andreas Paulus,^1,2,3^ Natascha Drude,^2,4^ Wouter van Marken Lichtenbelt,^5^ Felix M. Mottaghy,^2,3^ Matthias Bauwens^1,3;*^

^1^Department of Radiology and Nuclear Medicine, NUTRIM School for Nutrition and Translational Research in Metabolism, Maastricht University Medical Center, Maastricht, the Netherlands

^2^Department of Nuclear Medicine, University Hospital RWTH Aachen, Aachen, Germany

^3^Department of Medical Imaging, Division of Nuclear Medicine, MUMC, Maastricht, The Netherlands

^4^Department of Nanomedicine and Theranostics, Institute for Experimental Molecular Imaging, Uniklinik RWTH Aachen and Helmholtz Institute for Biomedical Engineering, Aachen, Germany

^5^Department of Nutrition and Movement Sciences, NUTRIM School for Nutrition and Translational Research in Metabolism, Maastricht University Medical Center, Maastricht, The Netherlands

Research School NUTRIM, Maastricht University, Universiteitssingel 50, 6229 ER Maastricht,

Netherlands

Corresponding Author: Dr. Matthias Bauwens

Email: [matthias.bauwens@mumc.nl](mailto:matthias.bauwens@mumc.nl)

Phone: +31 4338 74738

**Supplementary Information**

**
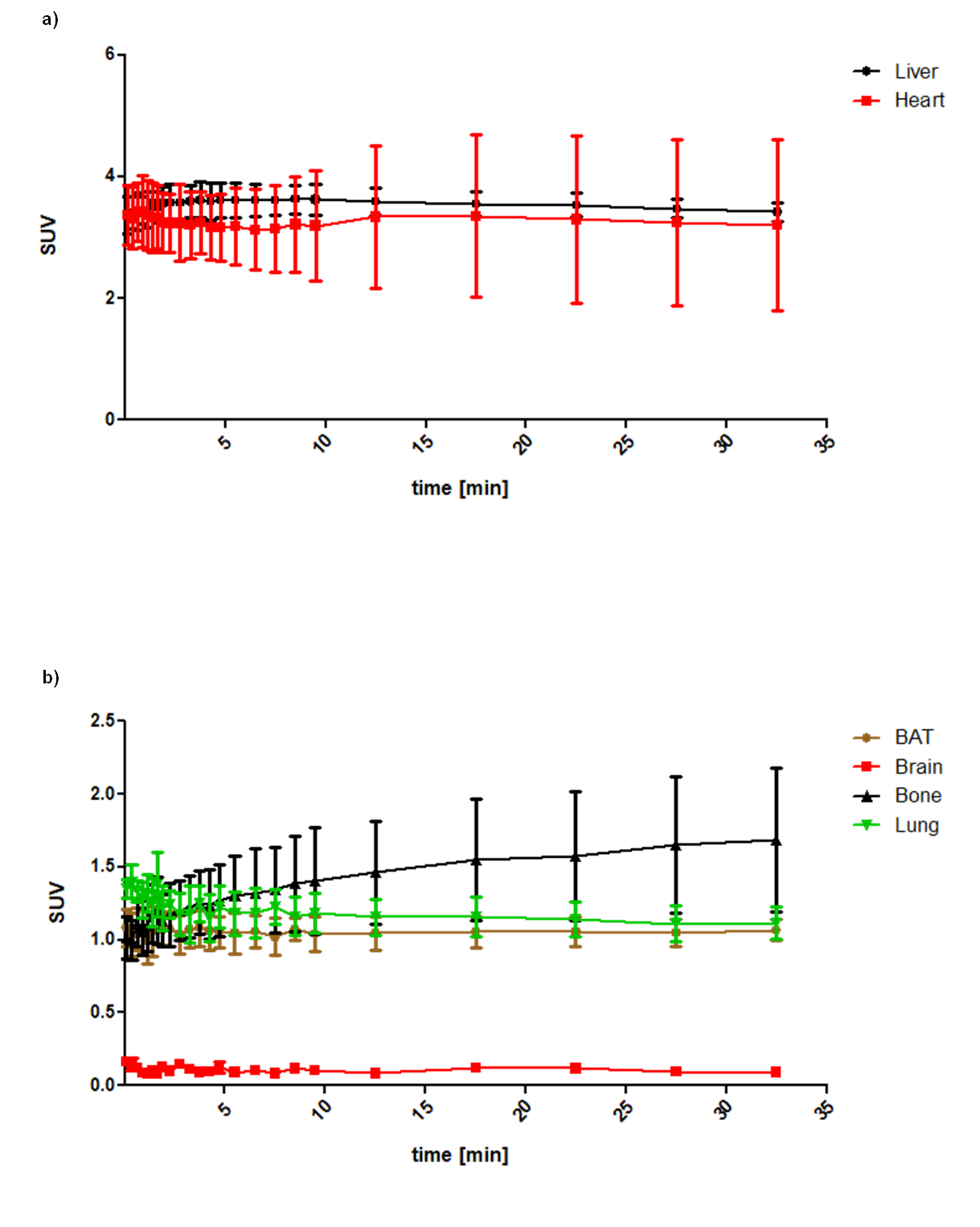
**

Fig S1: time activity curves in control mice housed 21 °C in a) liver and heart b) BAT, brain, bone, lung, exemplary for all other groups and sub-groups
